# Mammalian miRNA curation through next-generation sequencing

**DOI:** 10.3389/fgene.2013.00145

**Published:** 2013-08-02

**Authors:** Miguel Brown, Hemant Suryawanshi, Markus Hafner, Thalia A. Farazi, Thomas Tuschl

**Affiliations:** Laboratory of RNA Molecular Biology, Howard Hughes Medical Institute, The Rockefeller UniversityNew York, NY, USA

**Keywords:** miRNA, miRNA profiling, next generation sequencing, annotation, curation, RNA biogenesis, small RNAs

## Abstract

Characteristic small RNA biogenesis processing patterns are used for the discovery of novel microRNAs (miRNAs) from next-generation sequencing data. Here, we highlight and discuss key criteria for mammalian – specifically human – miRNA database curation based on small RNA sequencing data. Sequence reads obtained from small RNA cDNA libraries are aligned to reference genomic regions, and miRNA genes are revealed by their distinct read length and bimodal read frequency distribution, the predicted secondary structure of the deduced miRNA stem-loop precursor molecule, and, to a lesser degree, based on evolutionary conservation of small RNAs from other vertebrates. Properly curated miRNA databases are an important resource for investigators interested in miRNA biology, diagnostics, and therapeutics.

## INTRODUCTION

Rapid advances in sequencing technologies as well as the diagnostic and therapeutic promise of microRNAs (miRNAs) led to intense efforts in discovering and cataloging miRNA genes and establishing the miRBase repository to support their naming and archiving (Kozomara and Griffiths-Jones, 2011). Most of the abundant miRNAs have likely been discovered and characterized in humans and other mammalian species; however, less abundant and/or cell type-specific miRNAs remain to be cataloged. Furthermore, many entries recently placed in the repository may not be *bona fide* miRNAs. This is especially true of non-conserved and poorly expressed small non-coding RNAs (ncRNAs) that appear to have miRNA-like characteristics (Chiang et al., 2010). These annotation assignment errors are likely due to inconsistent standards in vertebrate miRNA curation. In this review, we outline miRNA biogenesis-guided criteria for the verification and validation of miRNAs derived from deep-sequenced small RNA cDNA libraries using the biological characteristics of miRNAs to create custom analyses. Many of the requirements discussed are shared with the standards established for plant miRNAs (Meyers et al., 2008). This review will focus on verifying canonical miRNAs, i.e., those miRNAs that are processed by both DROSHA/RNASEN and DICER1. The biogenesis of a small subset of miRNAs requires the spliceosome (mirtrons) or is only dependent on one of the two RNase III enzymes (Yang and Lai, 2011). Non-canonical miRNAs, such as hsa-mir-451 (hsa denotes “*Homo sapiens*”) produced without the help of DICER1, hsa-mir-320 processed without the help of DROSHA (Cifuentes et al., 2010) would not pass our criteria and therefore need to be reviewed using different methods.

### BIOLOGICAL CHARACTERISTICS OF miRNAs

#### miRNA biogenesis

MicroRNAs are small, regulatory ncRNAs 20–23 nucleotide (nt) long that repress gene expression predominantly by binding to the 3′UTR of target mRNAs in the form of ribonucleoprotein complexes that mediate mRNA destabilization (Krol et al., 2010). In mammalian cells, miRNAs are typically transcribed by RNA polymerase II as long pri-miRNA molecules from intergenic regions of the genome, but may also be derived from intronic and exonic regions of coding and non-coding genes (Kim et al., 2009). In the nucleus, canonical pri-miRNAs are processed by the RNase III DROSHA to produce a 60–100 nt precursor miRNA (pre-miRNA) hairpin molecule (Lee et al., 2003). The pre-miRNA is subsequently transported into the cytoplasm by Exportin 5 (XPO5) and further cleaved by another RNase III, DICER1 to generate a 20–23 nt miRNA–miRNA* duplex (Yi et al., 2003). The duplex is unwound by a helicase and the mature miRNA is incorporated into Argonaute proteins (AGO/EIF2C), while the miRNA* (read as miRNA “star”) is degraded (Hutvagner and Zamore, 2002; Haley and Zamore, 2004). Following formation of the RNA-silencing complex (RISC), the mRNA targets are recognized by partial sequence complementarity (Tuschl, 2004). The additional binding of GW182/TNRC6 proteins facilitates this recognition. Subsequently, the CAF1–CCR4 mRNA deadenylation complex is recruited to initiate mRNA degradation (Braun et al., 2013).

#### miRNA characteristics

Canonical, or otherwise known as prototypical, miRNAs are excised from a hairpin structure as a duplex consisting of miRNA and miRNA*. This process is linked to the following characteristics when assessing miRNA sequencing data: (1) Typically, miRNAs show presence of reads corresponding to the miRNA* sequence that are complementary to the mature miRNA forming a 2-nt 3′ overhang (Berezikov et al., 2010; Chiang et al., 2010). (2) Sequence analysis of mature miRNAs across different species reveals a strong bias for a U or an A at the 5′ position consistent with nucleotide-specific interactions in the MID domain of AGO proteins (Frank et al., 2010). (3) Processing variation of the 5′ end of miRNAs, is less frequent than variation of the 3′ end and thereby facilitates distinction between high-confidence miRNAs and likely turnover products from other abundant cellular RNAs. The seed sequence (position 2–8) of miRNAs is critical for mRNA regulation and required to nucleate the pairing between miRNA-loaded RISC and the target mRNA (Wang et al., 2008, 2009; Friedman et al., 2009).

#### Functional and genomic organization of miRNAs

**FIGURE 1:**
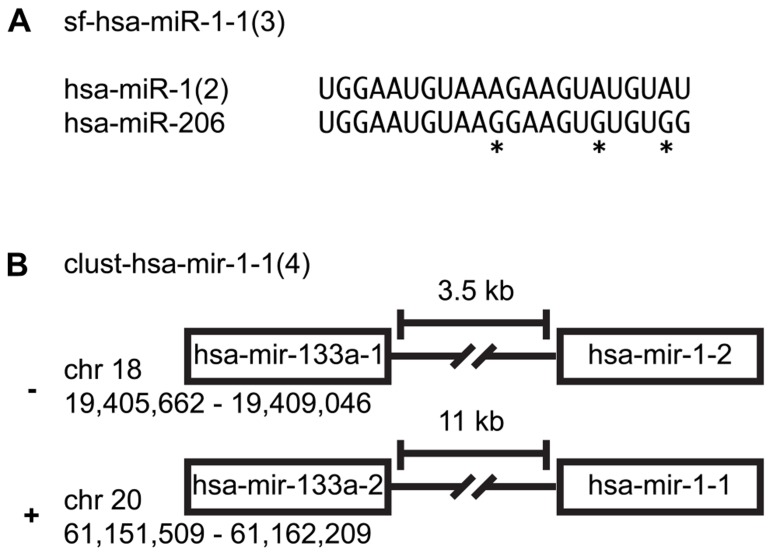
**miRNA family clusters. (A)** An example of a miRNA sequence family cluster. An asterisk indicates nucleotides that differentiate the two miRNAs. **(B)** An example of a precursor family cluster. While hsa-mir-1-1 and hsa-mir-1-2 are not on the same chromosome, their sequence composition makes them indistinguishable.

Many miRNAs share the same seed sequence that is used in mRNA targeting. Four hundred twenty-six of the 1,112 mature and miRNA*s can be organized into sequence families (Farazi et al., 2011, 2012). Naming of sequence families follows the proposed naming convention: sf-(three-letter species identifier)-miR/let-7 (lowest alpha-numeric member of group; number of members). For example, sf-hsa-miR-1-1(3) is a *H. sapiens* miRNA sequence family with three members: hsa-miR-1-1, hsa-miR-1-2, and hsa-miR-206 (Landgraf et al., 2007). miR-1 is a multi-copy miRNA expressed from two distinct genomic locations (**Figure 1A**; Farazi et al., 2012).

Two hundred ninety-nine of the 557 miRNA precursors can be organized into transcriptional units (cistrons, also known as genomic clusters). These cistronic miRNAs are typically located within 5 kb of each other in intergenic regions or within the same intron/exon and are co-transcribed and yield similar read counts for each member of a given miRNA precursor cluster (Farazi et al., 2012). Note that we merge miRNA cistrons containing members of the same multi-copy miRNA, since it is not easily ascertainable which copy is primarily responsible for the mature miRNA expression levels. Naming of genomic expression clusters follows this convention: cluster-(three-letter species identifier)-mir/let-7-(lowest alpha-numeric member of group; number of members). For example, cluster-hsa-mir-1-1(4) is a genomic cluster with four members: hsa-mir-1-1, hsa-mir-1-2, hsa-mir-133a-1, and hsa-mir-133a-2 (**Figure 1B**; Landgraf et al., 2007; Griffiths-Jones et al., 2008).

### TECHNICAL ASPECTS OF SMALL RNA SEQUENCING DATA ANALYSIS

#### Small RNA cDNA library preparation

**FIGURE 2:**
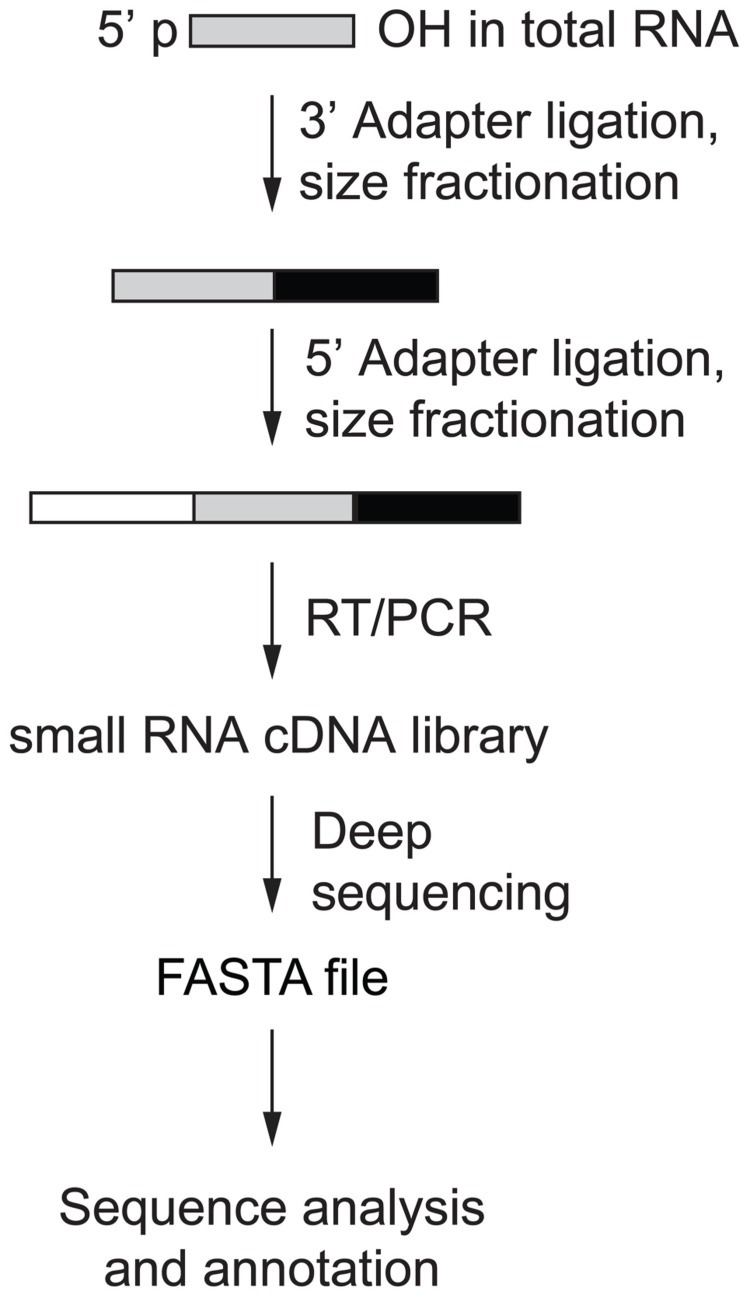
**Small RNA cDNA library preparation overview** (Hafner et al., 2011).

Human AGO-protein-associated RNAs are characterized by 5′-phosphate (p) and 3′-hydroxyl (OH) groups, and PIWI-protein associated RNAs (piRNAs) are additionally 2′-*O*-methylated at their 3′ ends (Saito et al., 2007). Protocols have been developed to take advantage of this chemical property and enrich for miRNAs and piRNAs over RNA turnover and hydrolysis products, arising especially from abundant rRNAs and tRNAs (Lau et al., 2001). Library preparation involves consecutive steps of adapter oligonucleotide RNA ligation and size selection that introduce primer-binding sites for subsequent reverse transcription (RT) and PCR amplification prior to deep sequencing (**Figure 2**; Hafner et al., 2011). The first step of the small RNA cDNA library preparation is to ligate the 3′ oligonucleotide adapter to the end of the sample RNA using an engineered T4 RNA ligase 2. Adapter-ligated sample RNA is then size-fractionated on denaturing polyacrylamide gels with the band in the size range of 19–24 nt plus 3′ adapter length excised and used for 5′ adapter ligation followed by another size fractionation step. Sequential ligation of two distinct adapters enables the retrieval of the original RNA strand orientation after sequencing and thereby represents a directional RNA sequencing method. The multiple purification steps following adapter ligation minimize the amount of insert-free adapter–adapter products otherwise observed in the final library. Finally, RT and PCR are performed and the resulting small RNA cDNA library is deep sequenced.

#### Annotation database choice

miRBase (Kozomara and Griffiths-Jones, 2011) is a well-organized repository of miRNA sequences complete with references, mature sequence definitions, as well as miRNA family grouping definitions found at http://www.mirbase.org. For approximately 200 species, miRNAs have been deposited or identified by homology searches as of release 19 and are searchable by species, chromosomal location, and sequence. The organization of miRNAs is systematic across species and the miRNA name is maintained if the miRNAs are orthologous, i.e., hsa-mir-107, a human miRNA and mmu-mir-107, a murine miRNA share the same name. Also, if a miRNA is homologous, meaning that there are 1- or 2-nt differences in different miRNAs in the same species, a sequential lower case letter is added. Additionally, a dash and an integer are added to indicate miRNAs with multiple genomic locations arising from gene duplication (multi-copy miRNAs). Naming of mature miRNA follows all of the conventions of the precursor with a couple of tweaks; first, all-lower case “mir” becomes “miR” to distinguish between gene and transcript, and secondly, if one arm is heavily favored over the other, the minor product has a * added at the end. If both arms are roughly equally expressed, then each product is likely to have a biologically important targeting effect and the transcripts corresponding to each arm receive -5p and -3p, respectively, according to their position relative to the 5′ end of the pre-miRNA. Usually, miRNA* are present at such low levels that they are unlikely to have significant regulatory effects. The naming conventions outlined above largely conform to those presented by Griffiths-Jones et al. (2008); however, release 19 miRBase dropped this naming convention, opting to name all mature products -5p/-3p.

While miRBase is generally a very useful resource, not all entries are reliable (Meng et al., 2012). Particularly, many of the recently added miRNAs are the result of misannotation, be it degradation products of other more abundant ncRNAs, or mismapping (poor read mapping evidence; Chiang et al., 2010). Misannotation is caused by over-reliance on prediction algorithms, secondary structure, and/or misinterpretation of the data due to an overly restricted frame of reference, especially when reviewing a combination of read coverage and secondary structure within a sequence window of inadequate short length. Further investigation of some of the recently added miRNAs points to lack of a biological function for these miRNAs (Chiang et al., 2010). There are some basic acceptance criteria, i.e., a minimum read count for mature miRNA, the prediction of a hairpin fold with overlap between mature and miRNA*, mapping to a limited number of locations in the genome or to an existing RNA of a different type. It is best to initially focus on miRNAs that fulfill these principles, and seek independent evidence for the importance of miRNAs that do not. Sequence reads falsely annotated as miRNAs can lead to incorrect experiment interpretation and false hypothesis generation.

#### Sequencing platform

The small RNA cDNA libraries sequenced by the Illumina platform typically yield 10–250 million reads of 50 bp, depending on multiplexing and the instrument used, which is sufficient for curation and profiling of miRNAs. Barcoding allows for acquisition of profiles for up to 20 samples using our approach, which would yield ~500,000 reads per sample (Farazi et al., 2012). A study using pooled synthetic miRNAs shows that variations in abundance by up to three orders of magnitude are quantified using this method (Hafner et al., 2011). Capturing rarely expressed miRNAs using increased sequencing depth, may not necessarily provide additional biological insight into miRNA-mediated mRNA regulation considering that such regulatory effects can only be observed experimentally for highly abundant miRNAs.

#### Read mapping

**FIGURE 3:**
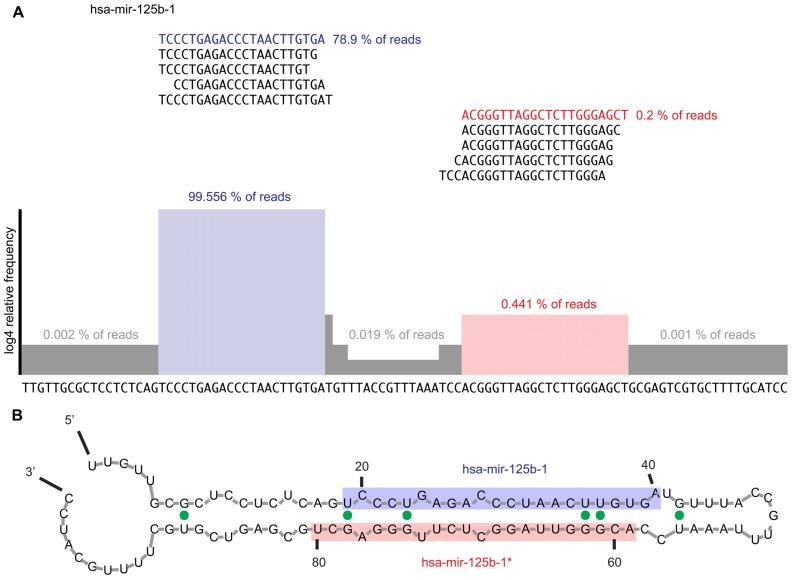
**(A)** Example of read alignment diagram for a prototypical miRNA. Displayed are the top five reads that mapped to the mature and miRNA* sequences, respectively. About 99.4% of all reads mapping to the precursor mapped to the mature arm while the top five reads mapping to the miRNA* arm only captured 0.4% of reads mapping to the precursor sequence. Highlighted in blue is the mature miRNA sequence; in red the miRNA* sequence. The histogram shows the read coverage over the precursor sequence, represented as log4 of relative read frequency. We suggest expanding the sequence 100 nt in either direction in order to assess for expression irregularities and ensure that the mature and miRNA* arms of the miRNA are correctly defined. Furthermore, all reads mapping at each error distance would be displayed. **(B)** Example of secondary structure prediction for mir-125b-1 precursor RNA. Mfold (Zuker, 2003) was used to generate a secondary structure prediction for each miRNA to confirm the foldback and overhang structure expected from a prototypical miRNA. The mature sequence is shaded blue, while the miRNA* sequence is shaded in red. The expected stem-loop structure and 2-nt 3′ overhang is observed. G–U wobbles are indicated by solid green dots.

The sequence read quality of small RNA cDNA libraries obtained from Illumina platforms allows for curation and verification of annotated and/or candidate miRNAs. The underlying methodical parameters of library preparation enrich for 5′ p and 3′ OH RNAs 19–25 nt (for miRNA) or 19–35 nt (for miRNA and piRNA) in length and minimize the impact of turnover products from abundant longer RNAs, e.g., tRNAs and rRNAs; however, because of concerns with miRBase’s continuing addition of miRNA candidates, mapping against other ncRNA databases is also recommended when curating sequences from assigning reads to miRBase-listed sequences. Annotation for other ncRNA sequences can be obtained from many sources, such as GenBank, ENSEMBL (Flicek et al., 2013), and UCSC Genome Browser (Meyer et al., 2013). For curation, we map reads allowing for a total of up to 2 bp mismatches, insertions, or deletions to accommodate for 3′ end A and/or U additions, as well as G to A changes resulting from dsRNA deamination. After directional mapping, read alignments to the precursor sequence are generated for visual inspection, and sorted by read counts. Separating the alignment views by number of errors allows for determination of the accuracy of the proposed precursor as well as the mature products. A distinct bimodal distribution of reads is seen 10–20 nt apart, with each peak ~22 nt wide (**Figure 3A**). Some recent miRBase entries include calls of the reverse complement of known miRNAs, some of which may, however, be caused by read mapping of sequencing errors of nearly palindromic miRNA and miRNA* sequence reads.

#### Expression profiling

Generation of the expression profile is the last step performed after sequencing and mapping the resultant reads from a small RNA cDNA library sequencing experiment. All of the reads mapping to the miRNA and miRNA* sequences are tallied and then analyzed for abundance and changes in expression. This can be achieved by using unsupervised hierarchical clustering methods. One such method developed uses a Bayesian framework (comparing the relative ratio likelihood of the model that the read frequency was the same between two samples for a given miRNA versus the model that they are different) to cluster samples and miRNAs by expression, measuring their distances, and then presenting the normalized results (log2 of the relative frequency) in a heatmap (Landgraf et al., 2007). Expression profiles of miRNAs provide crucial insight into interpretation of a deep sequencing experiment. The data obtained from various samples are visualized by creating a heatmap and using non-hierarchical clustering of miRNAs.

## DATA PROCESSING

**FIGURE 4:**
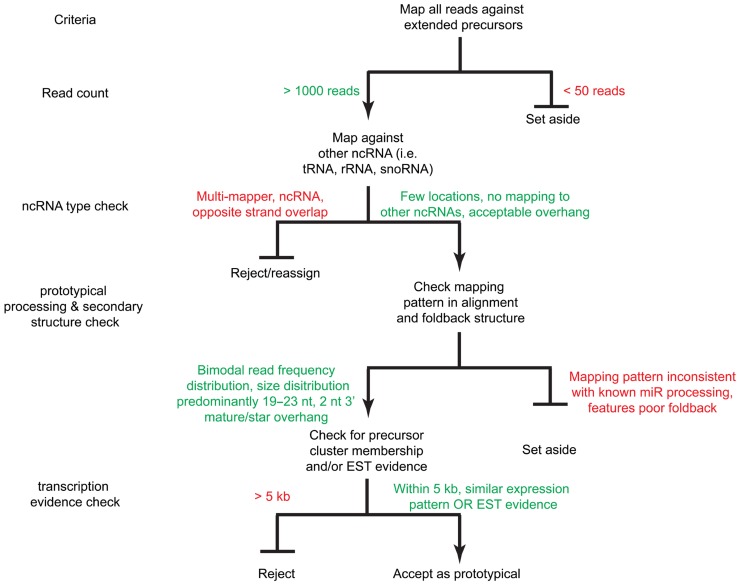
**A decision tree for miRNA acceptance criteria.** Sequence reads are mapped against the extended precursors as well as other ncRNA databases (i.e., tRNAs, snoRNAs, rRNAs, etc.). Reads with sufficient read count coverage, proper foldback and mature product overhang structure, as well as expression evidence by EST and/or genomic cluster membership are accepted as prototypical miRNAs.

**Figure 4** outlines our approach to miRNA curation using small RNA cDNA libraries. Review of existing miRNA definitions begins by downloading the sequences for all of the miRNAs in miRBase (current release 20) and mapping them to the human genome (NCBI release 37) using an aligner such as the Burrows–Wheeler aligner (BWA; Li and Durbin, 2009). Next, the flanking 100 nt to the annotated precursor are extracted allowing for better confirmation of the miRNA biogenesis pattern and identification of the miRNA* sequence, which is often not well-annotated in the miRBase entry for each miRNA. Precursors with greater than 30 genomic locations that are not part of an existing miRNA cistron are set aside as these are likely repetitive and/or low complexity regions that fortuitously collect reads (Landgraf et al., 2007). Next, we align pooled reads garnered from sequencing small RNA cDNA libraries allowing for up to two errors. The current sequencing technologies have an error rate of less than 1% (Minoche et al., 2011) so errors due to sequencing are rare; however, RNA editing and RT/PCR errors during library preparations warrant mapping at this relatively high error rate despite the short read length. Multi-mapping reads are allowed and are assigned to those sequences to which they map with the lowest number of errors. The number of genomic locations to which reads map is also used during miRNA curation.

### BASELINE ACCEPTANCE CRITERIA

We prioritize the analysis clusters of sequence reads by read counts in order to first discover or curate the higher expressed and more regulatory important miRNAs. In our curation of a collection of cDNA libraries comprising 2,000 human samples from healthy and disease tissue and cell lines with more than eight billion sequence reads, potential precursors with a read count of less than 50 were dismissed. Low read frequency miRNAs might either represent poorly expressed miRNAs or derive from cell types under-represented in a given sample. Review of the abundance within a specific sample rather than the pooled read collection may provide indicators to the value of these miRNAs in specific cell types or disease conditions. Preferably, acceptance of a new miRNA is supported by the existence of an ortholog and/or compelling experimental evidence. Candidate precursors accumulating more than 50 sequence reads are further inspected unless the non-redundant read count is below 5 preventing processing patterns to be recognized. Presence of reads for miRNA* sequence is important for identifying high-confidence prototypical miRNAs. Its presence provides strong evidence of processing by DROSHA and DICER1 participating in canonical miRNA biogenesis. The analysis of libraries prepared from cell lines and tissues deficient in factors required for miRNA biogenesis, e.g., knockouts for RNase III enzymes or the cofactor DGCR8, allows for discovery of miRNAs with unusual biogenesis, such as DICER1 or DROSHA independent miRNAs and mirtons (Yang and Lai, 2011). Additionally, we require further evidence of expression based on the following criteria: (1) whether the miRNA precursor falls within an existing defined genomic cluster of co-expressed miRNAs, (2) and/or whether expressed sequence tag (EST) evidence exists to support its transcription; EST coverage information can be obtained from the UCSC genome browser. **Table 1** illustrates application of these rules used for expression analysis in Farazi et al. (2011).

**Table 1:** Summary of miRNA review.

Total	Rejected				Accepted
miRNA precursors checked	Insufficient read count	No precursor cluster membership	Multi-map + ncRNA + complement	Mapping pattern/foldback	Prototypical precursors and biogenesis read patterns
1,045	282	28	63 + 33 + 4	78	557

* 
The starting number of human miRNA precursors reviewed from miRBase release 16 is represented in the first column while the last column represents miRNAs consistent with the acceptance criteria.*

### APPLICATION OF miRNA SEQUENCE CHARACTERISTICS

According to the canonical miRNA biogenesis process, two sharp peaks, of ~22-nt length, spaced 10–20 nt, are expected (**Figure 3A**). Using RNA folding software, such as RNAfold (Hofacker, 2003) or mfold (Zuker, 2003), secondary structure predictions can be made for putative miRNAs. Using the most frequent reads on the 5′ and 3′ ends, the mature/* or 5p/3p products can be assigned. Once the 5′ and 3′ products are ascertained, a 2-nt 3′ overhang of the mature products should be observed (**Figure 3B**). Reads mapping to precursors are crosschecked by mapping against other ncRNA databases before checking the foldback structure. For example, snoRNAs and tRNAs have similar transcript length and secondary structure. However, snoRNAs and tRNAs can be easily identified since they display unimodal read coverage across the transcript.

### REJECTION

**FIGURE 5:**
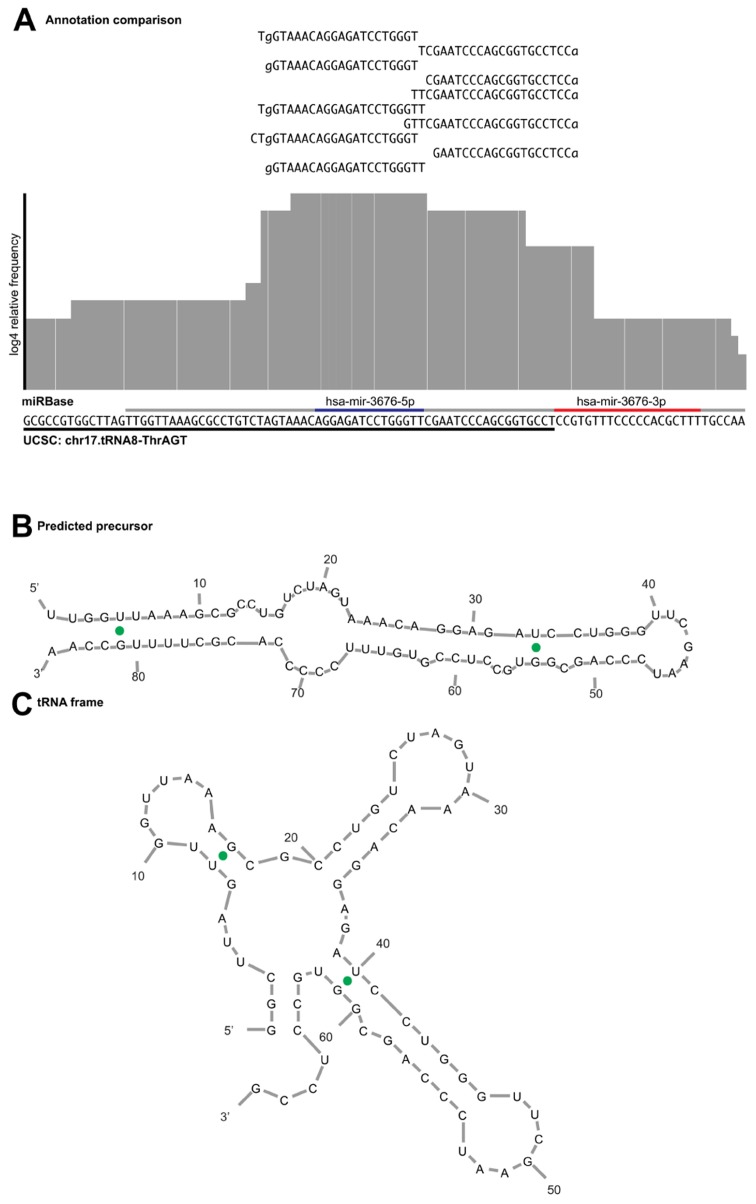
**Example of read alignment diagram for a miRNA not meeting the acceptance criteria. (A)** A similar view to **Figure 3** for putative mir-3676, including reads that map with one error distance to the precursor. Underlined in gray is the purported miRNA precursor, with the annotated mature and star sequences highlighted in blue and red, respectively. Underlined in black is the sequence annotated as tRNA pseudogene in the UCSC genome browser. The read evidence clearly supports a tRNA biogenesis pattern as the top reads map to that of the tRNA CCA 3′ terminus, rather than the bimodal distribution expected from a prototypical miRNA. **(B)** Predicted miRNA structure shows that the 5′ arm is shorter than what is expected for a mature miRNA product and does not produce the expected overhang. **(C)** If the sequence frame is shifted, the secondary structure prediction demonstrates a fold commensurate with that of a tRNA.

An example of a miRNA we would reject during curation is hsa-mir-3676. The precursor maps to a tRNA pseudogene, suggesting that the sequence reads derive from tRNA turnover products rather than specific miRNA processing. In addition, a view of the alignment using reads with one error reveals several flaws; the read mapping pattern does not demonstrate a clear bimodal distribution, there is no sufficient clearance between the 5′ and 3′ reads to accommodate a loop between the mature and miRNA* products (**Figure 5A**) and the folded stem-loop structure does not show the characteristic 3′ overhangs (**Figure 5B**). Note that the most frequent reads terminate with the tRNA-specific 3′ aminoacyl residue acceptor sequence “CCA.” If one were to adjust the start and end of the sequence of the predicted precursor and its predicted secondary structures, the read evidence would be indicative of a tRNA rather than a miRNA (**Figure 5C**). Lastly, **Table 2** illustrates the difference in mapping errors that can be seen between a high-confidence prototypical miRNA and a candidate miRNA that should be reclassified.

**Table 2:** Mapping error comparison.

Mapping errors	% of reads for hsa-mir-125b-1	% of reads for tRNA Thr AGT/hsa-mir-3676
0	80.59	16.32
1	15.72	44.64
2	3.68	39.04

*
This table shows the differences in mapping errors for the prototypical miRNA from **Figure 3** to the misannotated miRNA candidate from **Figure 5***

### ASSIGNMENT OF MATURE PRODUCTS AND NAMING CONVENTIONS

Once the 5′ and 3′ ends are determined, if the overall coverage of one arm exceeds 80% relative to the other, then the mature products are given mature/miRNA* designation, or otherwise, each arm is named 5p and 3p. While miRBase has decided to do away with such a distinction, we consider it important to maintain this nomenclature because it provides researchers with valuable functional information. Knowing that each arm is unevenly expressed, it is immediately apparent which arm should have a targeting effect when investigating miRNAs and targets of interest.

## FUNCTIONAL AND EXPRESSION-BASED ANNOTATION OF miRNAs – LAST STEPS

Those precursors that pass the criteria of being a canonical miRNA are checked for membership to an existing sequence family or are assigned a new one. No more than one symmetric mismatch or G–U wobble base pair or a 1-nt offset in the alignment at the 5′ end is permitted, in consideration that the seed sequence of the mature miRNA determines the regulatory target RNA interactions. If a newly identified miRNA is found to be part of an existing sequence family, then it is highly probable that it will target many of the same mRNAs that the other members do. Sharing of targets by members of the same miRNA sequence family in turn facilitates the identification of the predominantly regulated target mRNAs according to the overall abundance of miRNA families.

As discussed earlier, many miRNAs are organized cistronically and are processed from one long primary transcript. Thus, collapsing miRNA sequence reads belonging to genomic clusters yields a profile best representing miRNA transcriptional regulation. An added bonus is that the genomic cluster view is simpler to interpret than the single miRNA view, as well as gain greater statistical significance when conducting differential expression analysis. Much of the same information can be gleaned in terms of differential expression in between tissues and conditions with less clutter.

Using the workflow as prescribed is a powerful implementation in defining and verifying prototypical miRNAs. While the focus has been primarily on human miRNAs it can also be applied to other vertebrate systems.

## Conflict of Interest Statement

The authors declare that the research was conducted in the absence of any commercial or financial relationships that could be construed as a potential conflict of interest.
